# DNA Damage during G2 Phase Does Not Affect Cell Cycle Progression of the Green Alga *Scenedesmus quadricauda*

**DOI:** 10.1371/journal.pone.0019626

**Published:** 2011-05-16

**Authors:** Monika Hlavová, Mária Čížková, Milada Vítová, Kateřina Bišová, Vilém Zachleder

**Affiliations:** 1 Laboratory of Cell Cycles of Algae, Institute of Microbiology, ASCR, Třeboň, Czech Republic; 2 Faculty of Science, University of South Bohemia, České Budějovice, Czech Republic; Tulane University Health Sciences Center, United States of America

## Abstract

DNA damage is a threat to genomic integrity in all living organisms. Plants and green algae are particularly susceptible to DNA damage especially that caused by UV light, due to their light dependency for photosynthesis. For survival of a plant, and other eukaryotic cells, it is essential for an organism to continuously check the integrity of its genetic material and, when damaged, to repair it immediately. Cells therefore utilize a DNA damage response pathway that is responsible for sensing, reacting to and repairing damaged DNA. We have studied the effect of 5-fluorodeoxyuridine, zeocin, caffeine and combinations of these on the cell cycle of the green alga *Scenedesmus quadricauda*. The cells delayed S phase and underwent a permanent G2 phase block if DNA metabolism was affected prior to S phase; the G2 phase block imposed by zeocin was partially abolished by caffeine. No cell cycle block was observed if the treatment with zeocin occurred in G2 phase and the cells divided normally. CDKA and CDKB kinases regulate mitosis in *S. quadricauda*; their kinase activities were inhibited by Wee1. CDKA, CDKB protein levels were stabilized in the presence of zeocin. In contrast, the protein level of Wee1 was unaffected by DNA perturbing treatments. Wee1 therefore does not appear to be involved in the DNA damage response in *S. quadricauda*. Our results imply a specific reaction to DNA damage in *S. quadricauda*, with no cell cycle arrest, after experiencing DNA damage during G2 phase.

## Introduction

DNA damage is a threat to genomic integrity in all living organisms. Along with pyrimidine dimers caused by UV irradiation, double-stranded breaks are the most prevalent form of DNA damage. To cope with DNA damage, eukaryotic cells have developed a sophisticated network of proteins responsible for DNA damage recognition and repair [1], [2], [3], [4], [5]. The network impacts on cell cycle regulation and stops cell cycle progression if the DNA is damaged; this process is referred to as a DNA damage checkpoint. Depending on the timing of DNA damage, the checkpoint arrests the cell cycle at G1/S, S, or G2/M phases [6]. While genotoxic stress during G1 or G2 phase in mammals causes shorter or prolonged cell cycle arrest, genotoxic stress during DNA replication only transiently delays progression through S phase [2]. The G1 checkpoint is important in vertebrates but it is not conserved in simpler organisms like fission and budding yeast [7]. DNA damage during S and G2 phases activates a checkpoint that is highly conversed from fission yeast to humans and higher plants [8], [9]. Stress during S phase, such as depletion of deoxyribonucleotide pools or chemical inhibition of DNA polymerase, will cause stalled replication forks, leading to activation of the replication checkpoint [2]. The replication checkpoint utilizes the same network of proteins activated by DNA damage during G2 phase [2], [6], [8], [10], [11]. The network is very well characterized in mammals and yeasts [1], [2], [3], [4]. However, within the plant kingdom, the network is less well understood [5]. Throughout the three kingdoms there are two conserved proteins involved in the sensing and reaction to altered DNA structure. The generally accepted simplified paradigm states that double-stranded breaks activate ataxia telangiectasia mutated (ATM) kinase, while stalled replication forks and single stranded DNA damage activates ATM and Rad3- related (ATR) kinases [1], [2], [3], [4], [5]. In mammals, ATM/ATR block mitotic entry through activation of the downstream checkpoint homologs, 1/2 (Chk1/2) kinases, that inactivate the key mitotic activator, Cdc25 phosphatase, and activate the key mitotic inhibitor, Wee1 kinase [4]. The general paradigm holds true in higher plants. *Arabidopsis* mutants in *ATM* are hypersensitive to γ-radiation and methylmethane sulfonate [12] while mutants in *ATR* are hypersensitive to hydroxyurea, aphidicolin, and UV-B light [9]. ATM (and to lesser extent ATR) regulate transcription of a plethora of genes in response to ionizing radiation [5], [13]. The cell cycle genes induced in an ATM-dependent manner by ionizing radiation and double-stranded breaks include the B-type cyclin AtCYCB1;1 and the Wee1 kinase homolog, AtWEE1 [5], [13], [14]. This implies that effectors of the cell cycle block in response to DNA damage are similar in plants and animals, although plants lack Chk1/2 kinase.

**Figure 1:**
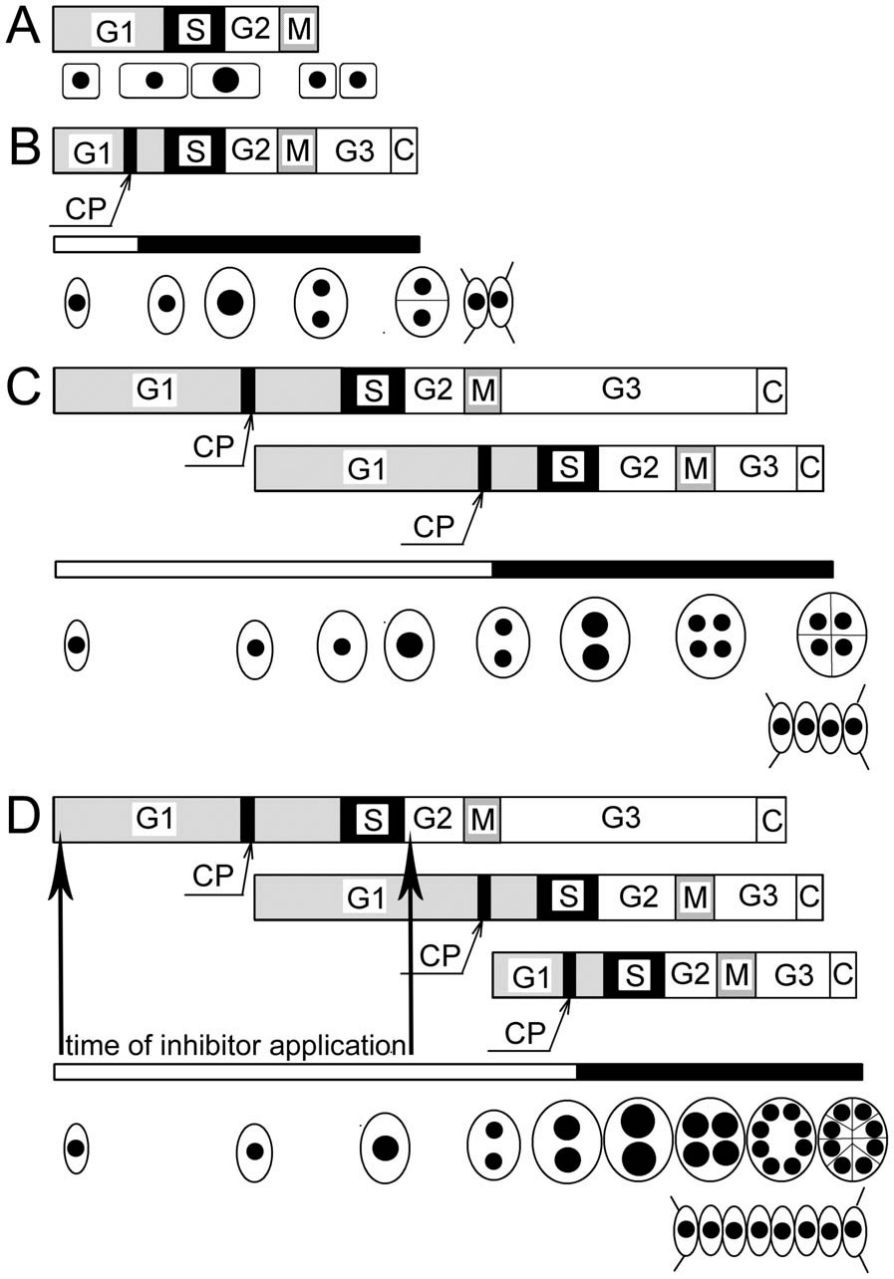
Schematic comparison of classical and multiple fission cell cycle of *S. quadricauda*. The classical model of the cell cycle [89] consisting of G1, S, G2 and M phases (**A**). G1 – growth phase, S – DNA replication, G2, M – nuclear division closely followed by cellular division occurs. Variants of cell cycle of *S. quadricauda* differ by the number of daughter cells produced (**B**, **C**, **D**). The cell cycle of *S. quadricauda* consists of G1, S, G2 and M phases as a classical cell cycle. However, nuclear (M) and cellular divisions (C) are separated by a G3 phase. During the G1 phase, cells attain a commitment point (CP), which triggers one round of S, G2, M, G3 and C sequence (a DNA-division sequence). Depending on the length of illumination and light intensity, the cells can attain one (**B**), two (**C**) or three (**D**) CPs for division into 2 (**B**), four (**C**) or eight (**D**) cells. At each CP, one DNA-division sequence corresponding to the entire classical cell cycle (represented by bars) starts. The individual DNA-division sequences run simultaneously and partially overlap. The processes before attainment of CP are light and growth dependent while the rest of the cell cycle after CP is light independent (indicated by white and black bars under the scheme of the DNA-division sequence). Schematic cells sizes are depicted during the cell cycle and the black spots illustrate the size and number of nuclei. Large black spots indicate the doubling of the DNA. The vertical arrows in **D** indicate the cell cycle phase when the inhibitors were applied. After Zachleder et al. [23], modified.

*Scenedesmus quadricauda* is a green monoplastidic alga (Chlorophyceae) forming coenobia [15]. The cells divide by multiple fission into 2, 4 or 8 daughter cells, as do other species of chlorococcal and volvocean algae. The cells in principle divide into 2^n^ daughter cells, where *n* is the number of doublings of daughter cell number [16], [17]. The cells grow during the G1 phase until they reach a critical cell size that is a prerequisite for attainment of the commitment point (CP), the formal equivalent of START in budding yeast or restriction point in mammals [18]. The processes starting after CP attainment do not require an external energy supply and, in the case of autotrophically grown cells, can be therefore performed in the dark. Attainment of CP enables one round of DNA replication, nuclear division, and cellular division (DNA-division sequence) (Fig. 1, [19], [20]). Depending on the growth rate, one or several CPs (up to three) are attained sequentially during the growth (light) phase of the cell cycle. Therefore up to three DNA replications, nuclear and cellular divisions can overlap during a single cell cycle, resembling the overlap observed during bacterial cell cycles (Fig. 1). The individual DNA-division sequences overlap each other but run independently. During the cell cycle the cells routinely become multinuclear; they also routinely undergo more than one round of DNA replication within a single nucleus (Fig. 1, [19], [20]). Moreover, since nuclear and cellular divisions are temporally separated during cell cycle progression, an additional gap phase, G3, was postulated to delimit nuclear and cellular divisions [21]. A detailed description of *S. quadricauda* cell cycle organization is provided elsewhere [20], [21], [22], [23], [24].

Progression through the cell cycle in *S. quadricauda* is regulated by cyclin-dependent kinase (CDK)-like kinases with histone H1 kinase activity in a similar fashion to other eukaryotes. There are at least two different complexes expressing CDK-like activity in *S. quadricauda*; one accompanies the attainment of CPs, and the other is able to interact with *suc*1 protein and its activity is specific for mitosis/es [25]. In the related green alga, *Chlamydomonas reinhardtii*, two types of major cell cycle regulating kinases, CDKA and CDKB, are encoded. In higher plants, CDKB is transcriptionally down-regulated in response to ionizing irradiation, but the transcription of CDKA is not affected [13]. In budding yeast, the transcription of *CDC28*, the homolog of CDKA, is not affected by DNA damage but its protein expression is increased [26]. *C. reinhardtii* also encodes a homolog of WEE1 kinase. WEE1 kinase in higher plants is transcriptionally up-regulated in response to both DNA replication and DNA damage checkpoints and it couples the cell cycle block in response to DNA damage with DNA repair [14] (Fig. S1).

Information on the reaction to DNA damage in *S. quadricauda* organism is scarce. The organism is moderately sensitive to UV radiation [27], which causes oxidative damage within the cells [28]. The effect of 5-fluorodeoxyuridine (FdUrd) on cell cycle progression is well characterized. FdUrd, an inhibitor of thymidylate synthetase, blocks nuclear DNA replication and consequently nuclear and cellular divisions [29], [30]. The effect of FdUrd resembles that of hydroxyurea and its application probably causes stalled DNA replication and consequently activation of ATR-related protein.

Zeocin is a DNA damage mimetic antibiotic causing DNA damage by cleaving both strands of the DNA molecule [31], [32], [33]. In other organisms, double stranded breaks activate ATM kinase [12], [14], [34], [35]. The double stranded breaks can be repaired throughout the cell cycle by an error prone mechanism, non-homologous end joining (NHEJ) or from late S to G2 phase by a high fidelity process, homologous recombination (HR) between sister chromatids [34]. The genome of *C. reinhardtii* encodes the genes necessary for both HR and NHEJ [36]. However, NHEJ seems to be the preferred repair mechanism in this organism [36], [37].

Trimethylxanthine caffeine is known to enhance the effect of many DNA damaging agents [38] in various organisms by alleviating G2/M checkpoint control and the connected DNA repair [39], [40], [41], [42]. However, for *S. quadricauda*, there is no clear understanding of the effect of DNA damage on the cells, the mechanisms controlling reactions to DNA damage, or the effect of DNA damage on cell cycle progression. This work is, to our knowledge, the first report describing the analysis of cell cycle progression in reaction to DNA damage in this organism.

We have studied the effects of two DNA metabolism-modifying compounds, zeocin and FdUrd, on cell cycle progression in *S. quadricauda*. The two compounds were also combined with caffeine, a chemical known to affect the cell's reaction to DNA perturbation. We show that the cells' response to zeocin differs depending on the timing of application. If applied before the onset of S phase, the cells firstly undergo a delayed S phase and then arrest permanently in G2 phase; the G2 phase block is partially abolished by application of caffeine. If applied in G2 phase, progression of the already running DNA-division sequences are not delayed or otherwise affected. The data indicate a unique response mechanism to DNA damage in *S. quadricauda*.

## Results

### Growth and cell cycle progression

#### Untreated cultures

**Figure 2:**
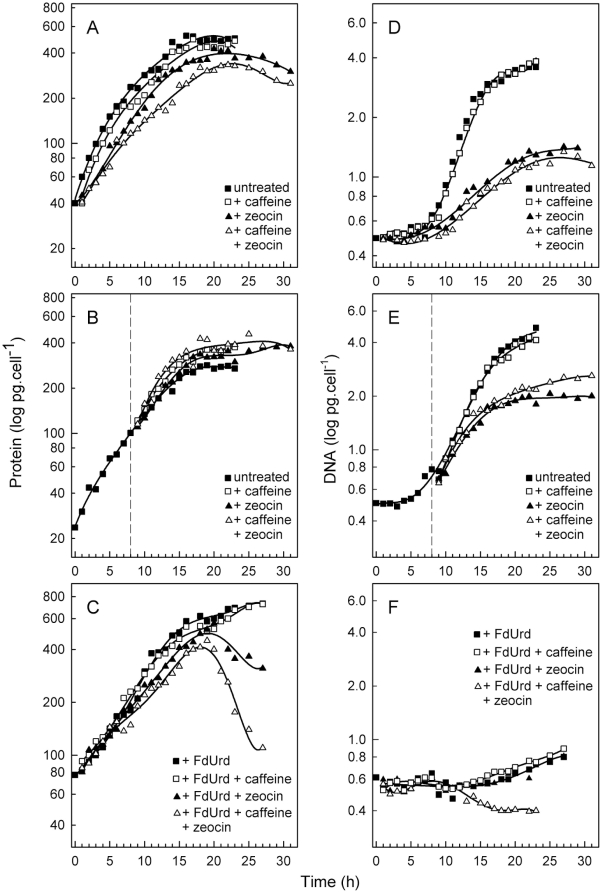
Increase in protein and DNA in synchronized populations of *S. quadricauda* cells. The left column of graphs (**A**, **B**, **C**) depicts changes in cellular protein in the untreated culture (solid squares), or in the presence of caffeine (open squares), zeocin (solid triangles) and caffeine + zeocin (open triangles) or the same compounds combined with FdUrd (**C**). The right column of graphs (**D**, **E**, **F**) shows the time course of DNA replication in the same cultures. The chemicals were present from the beginning of the experiment in **A**, **C**, **D**, and **F**. or added at the 8^th^ h (vertical dashed lines in **B**, **E**). The untreated culture was placed in the dark at the 15^th^ h in **A**. The mean values from two experiments are presented; variation about the means did not exceed 5% and were less than the diameter of the symbols.

Growth and cell cycle progression was followed in untreated cultures in order to set up a standardized platform on which the effect of the tested compounds could be compared. *S. quadricauda* cells were synchronized by three consecutive light/dark (15 h/9 h) cycles. Cell cycle progression under both light/dark cycle and continuous illumination was followed to show that there is virtually no difference between the two treatments. Cell growth was measured as changes in protein concentration per cell, similar patterns were also observed if cell size was measured (data not shown). During the light phase, total protein increased exponentially, about ten fold compared to the initial values (Fig. 2A, B).

**Figure 3:**
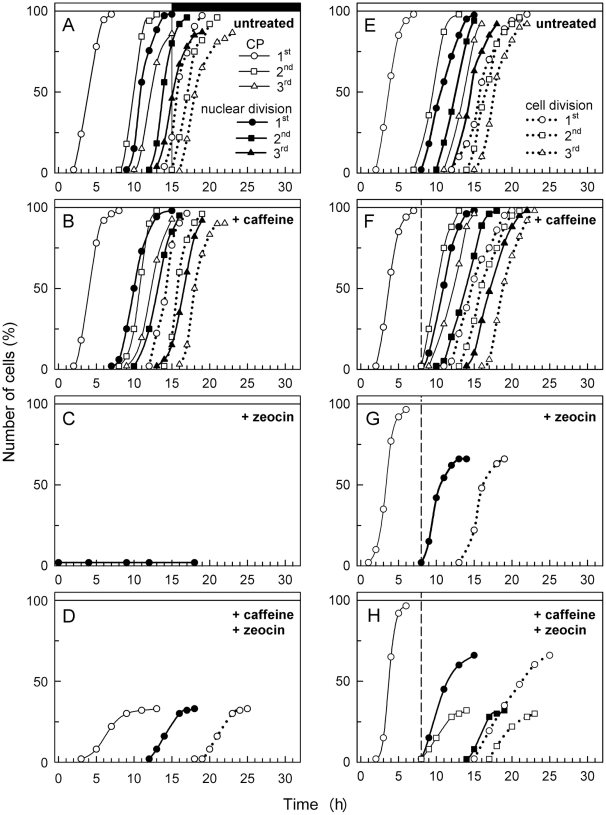
Cell cycle progression of untreated (A, E), caffeine-treated (B, F), zeocin-treated (C, G) and caffeine + zeocin-treated (D, H) synchronized populations of *S. quadricauda* cells. The graphs show the percentages of cells that attained CP (open symbols, solid lines, legend in **A**) (1^st^ CP (circles), 2^nd^ CP (squares), and 3^rd^ CP (triangles)), completed nuclear division (ND) (solid symbols, solid lines, legend in **A**) (1^st^ ND (circles), 2^nd^ ND (squares), and 3^rd^ ND (triangles)), and finished cell division (CD) (open symbols, dotted lines, legend in **E**) (1^st^ CD (circles), 2^nd^ CD (squares), and 3^rd^ CD (triangles)). The chemicals were present from the beginning of the experiment in **B**, **C**, and **D** or added at the 8^th^ hour of the cell cycle in (dashed vertical line) **F**, **G** and **H**. Light and dark periods are indicated by stripes above the panel and the transition between light and dark by the vertical solid line in **A**; all other cultures were incubated in continuous light. The mean values of 2 experiments are presented.

**Figure 4:**
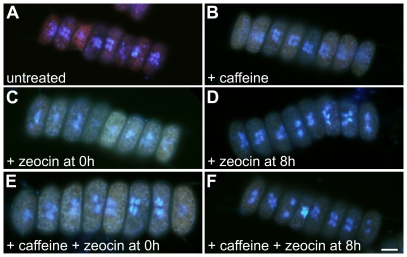
Photomicrographs of untreated (A), caffeine -treated (B), zeocin-treated (C, D) and caffeine + zeocin-treated (E, F) *S. quadricauda* cells from the 15^th^ (A, B) or 20^th^ (C–F) h of the synchronized cell cycle. The chemicals were added at the beginning of the cell cycle in **B**, **C** and **E**, or at the 8^th^ hour in **D** and **F**. Fluorescence photomicrographs comprise DAPI stained cells with nuclei. Bar = 10 µm (all photomicrographs).

An increase in cell size and protein is a prerequisite for cell cycle progression. Cell cycle progression continued during the light phase until the entire population of cells attained three CPs (for division into 8 cells) (Fig. 3A, E). Consequently, DNA levels increased about eight fold (Fig. 2D, E). Each DNA replication was followed by nuclear division, which finally resulted in 8 nuclei at the end of the cell cycle, division into 8 protoplasts, and the formation of an eight-celled daughter coenobium (Fig. 3A, E, 4A).

#### Cell survival in the presence of zeocin and caffeine

To determine the effective concentration of caffeine and zeocin, we tested the survival of *S. quadricauda* cells in the presence of different concentrations and combinations of zeocin and caffeine. The cells were grown in liquid culture under continuous light, serially diluted, and spotted onto a plate containing the drug/s. There was a clear distinction between the effects of zeocin and caffeine. Increasing concentrations of caffeine, from 1 mM to 3 mM, had little or no effect on cell survival. Increasing concentrations of zeocin above 4.4 µM significantly inhibited cell survival. Combining 4.4 or 8.8 µM zeocin with 1 to 3 mM caffeine increased cell survival compared to zeocin alone (Fig. S2). For more detailed experiments with synchronized cultures, a combination of 8.8 µM zeocin with 2 mM caffeine was chosen because under these conditions, the cells were killed by zeocin alone while this sensitivity was alleviated if caffeine was added.

Synchronized cultures were used to analyze the effects of these compounds on growth and cell cycle progression. The test compounds were applied either at the beginning of the cell cycle (G1 phase) or at the 8^th^ hour. At the 8^th^ hour, the cells had already attained the first CP (for division into two cells) (Fig. 3E). They all started, and mostly finished, the first replication of DNA (Fig. 2E). The first nuclear division did not proceed until the 10^th^ hour (Fig. 3E). At the 8^th^ hour, the cells were therefore in G2 phase of the first DNA-division sequence.

#### Caffeine-treated cultures

Caffeine is known to potentiate the effect of DNA damaging agents. It was applied (2 mM) either at the beginning of the cell cycle or from the 8^th^ hour of the light period.

Growth in the presence of caffeine was similar to that of untreated cultures (Fig. 2A, B), and was also similar to that of FdUrd-treated cultures if FdUrd and caffeine were combined (Fig. 2C).

Cell cycle progression of caffeine-treated cells was similar to that of untreated cells, i.e. they attained three CPs, attainment of each CP was followed by DNA replication and nuclear division, and the mother cells divided into 8 daughter cells (Fig. 3B, F). The application of caffeine affected neither growth nor cell cycle progression, irrespective of whether caffeine was added at the beginning of the cell cycle or at the 8^th^ hour.

#### Zeocin-treated culture

To study the effect of induction of double stranded breaks on cell cycle progression, a DNA damage mimetic antibiotic, zeocin, was used. Zeocin (8.8 µM) was applied either from the beginning of the cell cycle (G1 phase) or at the 8^th^ hour into the light period (G2 phase of the first DNA-division sequence – e. g. after the first S phase was completed and before nuclear division occurred) (Fig. 2E, 3E, G).

The cultures treated by zeocin from the beginning of the cell cycle grew slightly slower than untreated cultures (statistical difference p = 0.1), and final cell size was about 20% less (Fig. 2A). Growth was not affected if zeocin was applied in the 8^th^ hour of the light period (Fig. 2B).

The attainment of CPs and the following DNA-division sequences were significantly affected in zeocin treated cultures. In the cultures treated with zeocin from the beginning of the cell cycle, DNA levels were duplicated but the timing of DNA replication was delayed compared to an untreated culture (Fig. 2D). It could be assumed that the first CP was attained but of the committed DNA-division sequence (DNA replication-nuclear division-cell division), only DNA replication was allowed to be triggered and terminated. No nuclear or cellular divisions occurred in these cells (Fig. 3C); the cells were therefore blocked in G2 phase. The nuclei showed spread and dumbbell shapes (Fig. 4C).

If growing cells were allowed to attain their 1^st^ CP normally, but then treated with zeocin from the 8^th^ hr onwards, all further CPs were abolished (Fig. 3G). DNA was almost doubled at the time of treatment (Fig. 2E) and nuclear division did not proceed until the 9^th^ hour (Fig. 3E, G). The cells were therefore in the G2 phase of the first DNA-division sequence. About two thirds of cell population finished the nuclear division of the first DNA-division sequence (Fig. 3G). Some of the nuclei showed an aberrant spread structure, sometimes in the shape of a dumbbell, indicating a failure of nuclear division (Fig. 4D). Since DNA levels in the entire population quadrupled during treatment, it could therefore be assumed that the cells attained the 2^nd^ CP. Further progression through the second DNA-division sequence beyond DNA replication was blocked. Thus the second DNA-division sequence (allowed by attainment of the 2^nd^ CP) affected by zeocin prior to S phase was completely blocked in G2 phase (Fig. 3G).

Treatment with zeocin from the beginning of the cell cycle blocked cell cycle progression in G2 phase with duplicated DNA. However, of the cells treated with zeocin at the 8^th^ hour, two-thirds of them (66%) went on to a nuclear division only to become blocked at the next G2 phase.

#### Zeocin and caffeine treated cultures

Zeocin and caffeine were combined to test whether caffeine affects the response to DNA damage caused by zeocin. Similarly to single treatments, the combined treatment of zeocin (8.8 µM) and caffeine (2 mM) was applied either from the beginning of the cell cycle or from the 8^th^ h into the light period.

Cell growth was significantly slower (statistical difference p = 0.005) compared to untreated cultures if the treatment started at the beginning of the cell cycle (Fig. 2A). In contrast, cell growth was not affected if the treatment was applied at the 8^th^ hour of the cell cycle (Fig. 2B).

In cultures treated from the beginning of the cell cycle, 30% of the cells attained the 1^st^ CP, followed by nuclear and cellular divisions (Fig. 3D, 4E). Since the DNA levels were duplicated (Fig. 2D), analogous to zeocin-treated cells, it can be assumed that the population fully attained the 1^st^ CP. Therefore 70% of the cells were blocked in G2 phase. However, compared to zeocin-treated cells, 30% of the population were able to overcome the G2 block.

Before the application of caffeine and zeocin at the 8^th^ h of the cell cycle, the cells completely attained the 1^st^ CP (for division into two cells) (Fig. 3H). In contrast to zeocin treatment, the attainment of further CPs as well as progression through the DNA-division sequence was partially allowed in caffeine + zeocin-treated cells (Fig. 3 compare G and H). DNA levels were quadrupled (Fig. 2E), indicating that the entire population of cells attained the 1^st^ and 2^nd^ CPs. About 66% of the cells, similarly to zeocin-treated cultures, completed the first reproductive events and divided nuclei and cells into two (Fig. 3H). However, about 30% of the cells completed the second DNA-division sequence for division into two (Fig. 3H). In contrast, in the zeocin-treated culture, none of the cells completed the 2^nd^ DNA-division sequence (Fig. 3G). This indicates that about 30% of the cells in the zeocin and caffeine treatment were able to overcome the G2 phase block imposed by zeocin in the 2^nd^ DNA-division sequence. Similarly to the zeocin-treated cells, some of the nuclei had aberrant structures (Fig. 4F).

The data suggest that caffeine, in the presence of DNA damage, enables partial (30%) overriding of the DNA damage-induced G2 phase cell cycle block.

#### FdUrd-treated cultures

To inhibit nuclear DNA replication, 5-fluorodeoxyuridine (FdUrd) (25 µg/ml) was used. Cells in the presence of FdUrd showed similar protein accumulation to that of untreated cultures (Fig. 2 compare A and C) but nuclear DNA replication was inhibited (Fig. 2F). The slight increase in total DNA in the presence of FdUrd is probably due to chloroplast DNA replication, which is unaffected by this treatment (Fig. 2F, [29], [30]). The application of FdUrd inhibited nuclear division while growth remained unaffected. In the presence of FdUrd, attainment of the CP could not be followed by incubating the cells in darkness because DNA replication was blocked and neither nuclear nor cellular division could be observed. When FdUrd was removed prior to dark incubation, attainment of the CP was similar to that observed in Fig. 3A, E (data not shown), which indicated that the cellular state corresponding to CP was attained but the following committed events including DNA replication were prevented.

To identify possible additive effects, FdUrd was combined with zeocin and/or caffeine. Growth in the presence of FdUrd + zeocin as well as FdUrd + zeocin + caffeine was initially comparable to that of FdUrd treated cultures (Fig. 2C). However, protein degradation occurred later (Fig. 2C). This was indicative of severe strain to the cells, some of which most probably died. This was confirmed by evidence of DNA degradation in FdUrd + zeocin + caffeine treated cultures (Fig. 2F) and by approximately a 50% decrease in the amount of chlorophyll in treated cells compared to controls. No cell cycle progression occurred in the cells.

The treatment with FdUrd caused separation of growth processes (protein synthesis, cell size growth and attainment of CP) from progression through DNA-division sequences, due to inhibition of DNA replication. The combination of FdUrd and zeocin, and more so FdUrd + zeocin + caffeine, clearly strained the cells compared with FdUrd treatment alone.

### Cell cycle regulators in the presence DNA perturbing chemicals

#### Protein levels

To identify mechanisms controlling cellular reactions to DNA damage, we measured the protein levels of several cell cycle regulators using specific antibodies in Western blots. We chose three crucial cell cycle kinases: CDKA, CDKB and WEE1.

**Figure 5:**
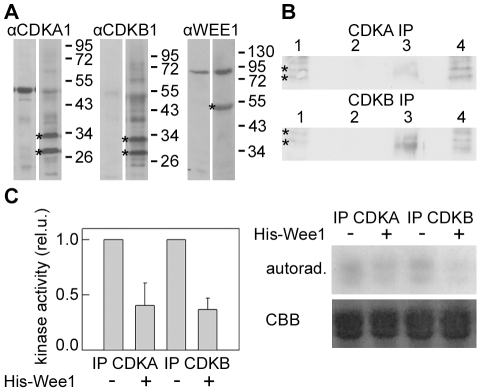
Specificity of antibodies against CrCDKA1, CrCDKB1 and CrWEE1 (A), specificity of the immunoprecipitation by anti CDKA and CDKB antibodies (B), and kinase activities of CDKA and CDKB immunoprecipitates upon phosphorylation by WEE1 (C). Protein extracts from mitotic cells separated by 12% SDS-PAGE and blotted onto Immobilon P were detected with pre-immune sera (left panels) and with sera raised against *Chlamydomonas reinhardtii* CDKA1, CDKB1 or WEE1 specific peptides (right panels). Specific bands of approximate molecular weight of 28 and 34 kDa (CDKA, CDKB) and 55 kDa (WEE1) are depicted by asterisks (**A**). Proteins were immunoprecipitated from mitotic extracts with either anti-CDKA or anti-CDKB antibody and detected using peroxidase-conjugated second antibody; lane 1, total mitotic protein extract; lane 2, immunoprecipitate from 50 µg total protein; lane 3, immunoprecipitate from 250 µg total protein; lane 4, flow through after immunoprecipitation; asterisks depict the specific bands (**B**). CDKs were immunoprecipitated by anti-CDKA or anti-CDKB antibodies from mitotic extracts and incubated with His-Wee1; histone H1 phosphorylation was assessed for each respectively. The graph quantifies the amounts of histone H1 phosphorylation normalized against the amount of histone H1 present; the mean of 2 different experiments is presented and a representative image is presented on the right (**C**). CBB – Coomassie Brilliant Blue stained gel. A representative image of at least 2 independent experiments is presented in **A** and **B**.

**Figure 6:**
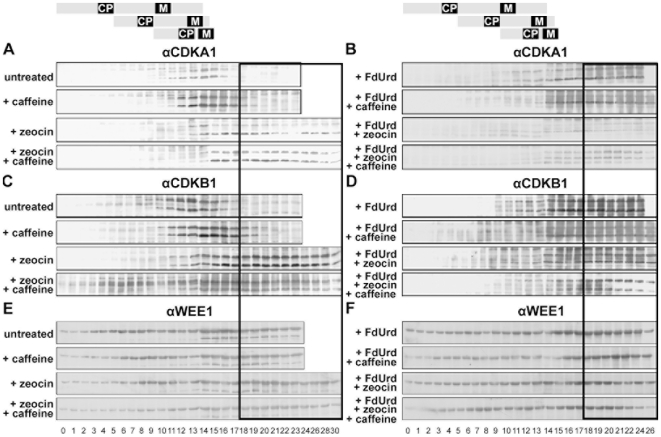
Protein levels of CDKA (A, B), CDKB1 (C, D) and WEE1 (E, F) kinases in synchronized populations of *S. quadricauda* cells. The cells were grown in the absence or presence of caffeine, zeocin or FdUrd and their combinations from the beginning of the cell cycle as indicated. The panels depict protein levels detected by specific antibodies. An equal volume of protein extract per cell was loaded. The main differences between untreated and treated cultures are depicted in the boxed section. A schematic representation of cell cycle progression in the untreated culture is indicated on the top. A representative image of 2 experiments is presented.

For the assays (CDKA, CDKB, Wee1 kinases), we used antibodies raised against specific peptides from the related green alga *C. reinhardtii*. The anti-Wee1 antibody reacted with a protein of apparent molecular weight 55 kDa (Fig. 5A). The antibodies against CDKA and CDKB cross-reacted with 2 bands of the same apparent MWs (28 kDa and 34 kDa) (Fig. 5A). Each antibody immunoprecipitated both proteins from a cell lysate; anti-CDKA precipitated approximately equal amounts of both proteins, whereas the anti-CDKB precipitated predominantly the 28 kDa protein and only a small amount of the 34 kDa protein (Fig. 5B). Therefore we assume that the 34 kDa band corresponds to CDKA while the 28 kDa band corresponds to CDKB. The accumulation of CDKA and CDKB occurred at a similar rate over the experiment. In all cultures, these proteins were detectable from the 11^th^ or 12^th^ to 16^th^ hour of the cell cycle. In untreated cultures, the maximum amount of CDKA was present from the 14^th^ to the 15^th^ hour. CDKB maximum was broader and occurred from the 10^th^ to the 16^th^ hour. The amounts of both of the kinases decreased to below detectable levels at the 17^th^ hour (Fig. 6A, C,. S3A, C). In caffeine-treated cultures, the kinetics of CDKA accumulation was similar to untreated cultures. CDKB protein was present from the 10^th^ to the 19^th^ hour. The peak level of protein was slightly shifted to later time points when compared with untreated cultures (Fig. 6A, C, S3A, C). In zeocin and caffeine + zeocin treated cultures, the CDKs appeared similarly to the untreated culture but they persisted throughout the entire experiment (Fig. 6A, C, S3A, C). Similarly, they persisted in FdUrd and FdUrd + caffeine treated cultures (Fig. 6B, D, S3B, D). In FdUrd + zeocin and FdUrd + zeocin + caffeine treated cultures, the levels of CDKs were close to the detection limit but did not decrease until the end of the experiment (Fig. 6B, D, S3B, D).

Wee1 was present throughout the entire cell cycle for all treatments. In untreated cultures, it increased at the 6^th^ to 8^th^ h of the cell cycle, at the time of the first nuclear division. It increased again at the 13^th^ to 16^th^ h of the cell cycle when the 2^nd^, 3^rd^ and 4^th^ nuclear divisions occurred (Fig. 6E, S3E). Both the protein level and the rate of accumulation were similar in the presence of caffeine (Fig. 6E, S3E). With zeocin alone, Wee1 begun to increase later, at the 8^th^ h, reached 2 peaks, at the 10^th^ and 13^th^ h, and was maintained high until the 23^rd^ h when it began to decrease (Fig. 6E, S3E). Wee1, in the presence of caffeine + zeocin, started to increase at the 11^th^ h and was maintained thereafter (Fig. 6E, S3E). Generally, Wee1 protein level was higher in the presence of FdUrd than in its absence (Fig. 6 compare E and F, S3E, F). In FdUrd treated cultures, Wee1 peaked twice at the 6^th^ and 11^th^ h and was maintained at this level thereafter (Fig. 6F, S3F). In FdUrd + caffeine treated cultures, Wee1 started to increase later, at the 16^th^ h, reached a peak at the 24^th^ hour and then decreased (Fig. 6F, S3F). In the presence of FdUrd + zeocin, and FdUrd + zeocin + caffeine, the rate of Wee1 accumulation was similar to that in cultures treated with FdUrd alone, although total Wee1 protein was lower (Fig. 6F, S3F).

The protein levels of both CDKA and CDKB kinases were stabilized in the presence of zeocin. In contrast, Wee1 was not affected by the presence of zeocin, FdUrd, caffeine or their combinations.

### Kinase activities

Kinase activity assays were performed to determine how changes in protein levels were related to the activities of CDKA and CDKB, as well as to see if there were changes in kinase activities that did not relate directly to protein levels.

An indirect kinase activity assay was performed to check whether the kinase activities associated with the antibodies could reflect changes in the activity upon phosphorylation with Wee1. The kinase complexes immunoprecipitated from 250 µg of total mitotic cell protein extract were pre-incubated in the presence or absence of 250 ng of purified His-tagged *C. reinhardtii* Wee1 prior to histone kinase assay. In the presence of His-CrWee1, kinase activities of both immunoprecipitated kinase complexes were reduced to about 40% of kinase activities in untreated cultures (Fig. 5C). The extent of inhibition was in agreement with the known effect of mammalian Wee1 phosphorylation [43], [44], [45], [46], and indicated that both cyclin-dependent kinases are inhibited by Wee1 to some extent.

In these experiments, kinase activities associated with anti-CDKA and anti-CDKB antibodies were assayed after immunoprecipitation. For each kinase assay, the same volume of total protein extract made from a constant number of cells was used; for immunoprecipitation, 20 µg of total protein from cells at the beginning of the cell cycle, and up to 500 µg of total protein from cells at the end of the experiment, were used. The kinase assay results therefore reflect changes in kinase activity per cell during the experiment. Both immunoprecipitates showed similar changes in kinase activity, as expected, since both antibodies immunoprecipitated CDKA and CDKB.

**Figure 7:**
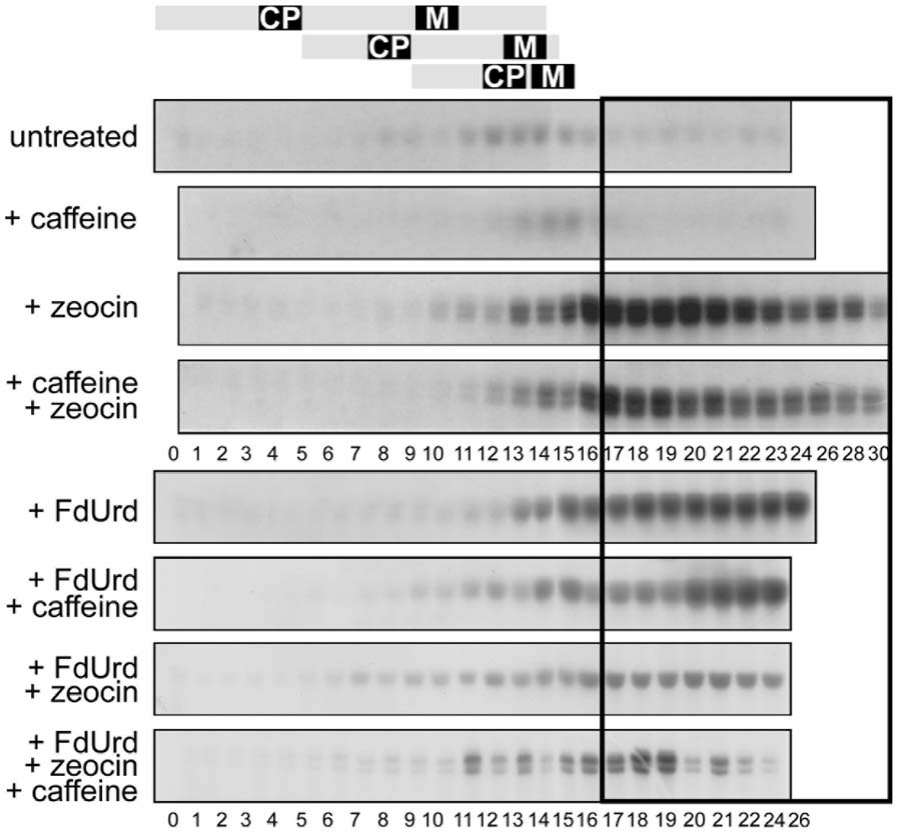
Kinase activities of anti CDKA immunoprecipitated kinases in synchronized populations of *S. quadricauda* cells. The cells were grown in the absence or presence of caffeine, zeocin or FdUrd and their combinations from the beginning of the cell cycle as indicated. The panel depicts kinase activities towards histone H1 as a substrate. The main differences between untreated and treated cultures are depicted in the boxed section. A schematic representation of cell cycle progression in the untreated culture is indicated on the top. A representative image of 2 experiments is presented.

In untreated cultures, the maximum kinase activity occurred at 12 to 16 h of the cell cycle for CDKA and the 12^th^ to the 14th hour for CDKB. It correlated with the 2^nd^ and 3^rd^ nuclear divisions and with cell divisions (Fig. 7, S4). Kinase activity in caffeine-treated cultures was lower than in untreated cultures, however, the timing of the changes in kinase activity was the same, being associated with nuclear divisions in both cultures (Fig. 7, S4). The kinase activities in zeocin and caffeine + zeocin treated cultures were higher compared to both untreated and caffeine treated cultures (Fig. 7, S4). Kinase activities increased with about the same timing as in untreated cultures but reached higher values and persisted at this elevated level for at least several hours until they decreased towards the end of the experiment (Fig. 7, S4). Similar results were found for FdUrd treated cultures; kinase activities started to increase at the time when nuclear division would occur in an untreated culture, and then persisted at an elevated level for the rest of the experiment (Fig. 7, S4). Cultures treated with FdUrd + caffeine displayed a 2-step increase in kinase activities. Firstly they increased to moderate levels approximately at the time when nuclear division should occur, then another increase in kinase activities occurred reaching levels similar to those in FdUrd treated cultures (Fig. 7, S4). In contrast, the kinase activities in both FdUrd + zeocin and FdUrd + zeocin + caffeine treated cultures were much lower than in the presence of FdUrd or zeocin alone, or caffeine + zeocin. Notably, the timing of increased kinase activity was similar to that for all cultures (Fig. 7, S4).

Overall, the results suggest that treatment with zeocin, FdUrd and their combinations caused an elevation in mitotic kinase activity, although the timing of increased kinase activity remained the same as in untreated cultures.

## Discussion

The DNA damage checkpoint is a mechanism that delays or arrests cell cycle progression in response to DNA damage. It is conserved in all eukaryotes from fission and budding yeasts [47], [48], [49], mammals [50], [51], higher plants [41], [42] to green algae [52]. Here, we showed that in the green alga, *Scenedesmus quadricauda*, such a mechanism could be observed only if DNA is affected prior to S phase; if DNA is damaged during G2 phase, no cell cycle progression delay or arrest could be observed in two thirds of the cells (Fig. 3C, G).

In *S. quadricauda* cultures, we have observed that addition of zeocin, an antibiotic causing double stranded breaks in DNA [31], [32], [33], prior to S phase, caused a delay in S phase, but it did not prevent DNA replication itself. In contrast, nuclear and cellular division did not occur, indicating that the cells were blocked in G2 phase. The same treatment during G2 phase, did not affect progression within the corresponding DNA-division sequence. DNA replication, nuclear division and cell division each occurred with the same timing as in untreated cells (Fig. 3). However, other DNA-division sequences that were affected prior to G2 phase were not completed and the cells were again blocked in G2 phase. The inability to block cell cycle progression in G2 phase could have been caused by lack of zeocin uptake during G2 phase, but because the S and G1 phases of the next DNA-division sequences within the same cells were affected by the treatment, this suggests that cell uptake is not a problem. Alternatively, G2 phase cells could be less sensitive to the treatment due to some DNA protective mechanism, or simply because of the absence of DNA damage sensing or DNA damage response during this phase. We do not know how much DNA damage was caused by the dose of zeocin that we used. The effect of zeocin on DNA is well characterized [31], [32], [33], and similar concentrations of zeocin were able to cause DNA damage in the green alga *C. reinhardtii* [53], and in a plant, *Arabidopsis thaliana* [14]. A single DNA break is enough to induce the DNA damage response pathway in yeast [54], [55], [56] and in mammals [57], [58], [59]. We therefore assume the observed effect on *S. quadricauda* cells is due to DNA damage caused by zeocin. The double stranded breaks caused by zeocin could be repaired either by NHEJ or HR. The DNA damage caused prior to S phase could be only repaired by an error prone NHEJ while damage during G2 phase, when the DNA was duplicated, it could be repaired either by NHEJ or by HR utilizing recombination between sister chromatids. It is not known which of the processes is used in *S. quadricauda*. In a related green alga, *C. reinhardtii*, NHEJ is the preferred repair mechanism [37] although both NHEJ and HR could be used [36].

The sensitivity towards zeocin was partially (30% of the cells) reduced in the presence of low concentrations of caffeine, an agent that enables an override of DNA replication and DNA damage checkpoints [39], [40], [41], [42], [60], [61] due to its specific inhibitory effect on ATM and ATR kinases [62], [63], [64]. The application of caffeine specifically overcame the G2 phase block caused by application of zeocin, while the S phase delay remained unaffected (Fig. 2, 3). On contrary, caffeine had no effect if combined with FdUrd. We did not observe any DNA replication or any attempted aberrant nuclear or cellular divisions. The presence of FdUrd completely blocked not only DNA replication but also the consequential cell cycle processes and the block could not be overcome by caffeine. In the survival tests, caffeine increased the survival of zeocin treated cells. This could be because the DNA damage caused by zeocin is not lethal if the arrest mechanism (G2 block) is removed by caffeine. A similar effect could be seen in budding yeast *cdc13* mutants that normally arrest at late S/G2 phase [65]. However, this arrest is abolished in the *cdc13 rad9* double mutants that combine the *cdc13* mutation with a mutation in the DNA damage checkpoint. The double mutants undergo several divisions in the presence of damage that is able to cause arrest but is not lethal [65]. By analogy, in mammalian cells defective in p53, the cells are more UV-resistant because they fail to arrest in the presence of DNA damage [66]. Although this process will eventually lead to greater genome instability, the cells are capable of division.

The cell cycle regulators examined here showed distinct behaviors in the presence of DNA perturbing chemicals. In the presence of FdUrd or zeocin, the levels of CDKA and CDKB proteins were maintained, and this was supported by high and persisting kinase activities in those cells. A similar effect of FdUrd in maintaining both high mitotic kinase activity and a cell cycle regulator, cyclin B, was reported previously in *S. quadricauda* [67]. This would indicate that, in *S. quadricauda*, CDKs are activated independently of the cell cycle progression (DNA replication block). However, progression through the cell cycle is blocked in these cells indicating that active CDKs might be somehow (spatially) prevented from carrying out substrate phosphorylation. Surprisingly, CDKA kinase activity reached its peak before the CDKA protein reached its maximum. This was probably caused by active CDKB that was also present in the immunoprecipitate. Wee1 was maintained at high levels in the presence of zeocin, caffeine and FdUrd. However, it is not clear whether this protein was enzymatically active since its target CDK maintained high kinase activity whereas, in the presence of Wee1, CDK activity would be expected to decline. Assessment of tyrosine phosphorylation of CDK may help clarify this point. Unfortunately, we were unable to do this because the specific antibodies we tested gave non-specific reactions. High CDK kinase activity in the absence of Wee1 mediated tyrosine phosphorylation would indicate that *S. quadricauda* uses a potentially novel way to ensure a cell cycle block in the presence of DNA damage. This could be related to its specific division plan by multiple fission (see below).

The data presented here indicate that the green alga, *S. quadricauda*, does not respond by a cell cycle progression block to the presence of DNA perturbing chemicals applied during the G2 phase, although it does so if the same chemicals are applied prior to S phase. Similarly, a G1 phase checkpoint in budding yeast is differently sensitive to DNA damage than S/G2 phase checkpoints [68] and at low levels of DNA damage it activates only a truncated version of DNA damage signaling response [69]. Cell cycle progression in the absence of DNA repair following the DNA damage will lead to division with damaged DNA. Several consecutive DNA-division sequences partially overlap during a single cell cycle of *S. quadricauda*; these sequences are in other cell cycle phases than G2 and would therefore respond to DNA damage with delayed S phase and a G2 phase block, similar to that occurring if the chemicals were applied at the beginning of the cell cycle. This means that the divided cells would be diploid and therefore could repair DNA with both error prone NHEJ and high fidelity HR mechanisms. It is not known whether, and to what extent, HR is used in *S. quadricauda* to repair double stranded breaks. It is not a preferred repair mechanism in the related green alga *C. reinhardtii* [37], however, it is the main repair mechanism in other unicellular organisms like yeast [70] and in mouse embryos [71]. As is mentioned above, p53 is necessary for mammalian cell cycle arrest in response to DNA damage, and its deletion leads to cell division even in the presence of damaged DNA. Interestingly, deletion of p53 alone is also able to partially induce pluripotent cells from mouse embryonic fibroblasts and improves induction of pluripotent cells in other conditions [72]. This implies that there could be some connection between failure to arrest cell cycle in response to DNA damage and the ability to dedifferentiate and divide. This is further supported by the absence of a DNA damage checkpoint during the rapid first embryonic division in *Xenopus laevis*, consisting of only S and M phases [73], [74], [75].

## Materials and Methods

### Experimental organisms, culture growth conditions, cell cycle synchronization and analysis

The chlorococcal alga *Scenedesmus quadricauda* (Turp.) Bréb., strain Greifswald/15 was obtained from the Culture Collection of Autotrophic Microorganisms kept at the Institute of Botany, Třeboň, Czech Republic. The cultures were synchronized by at least two cycles of alternating light and dark periods (15:9 h). The suspensions of synchronous cells (10^6^ cells/ml) were grown at 30°C in inorganic nutrient medium [76], aerated with air containing 2% (v/v) CO_2_ and illuminated by OSRAM L36/41 fluorescent tubes; the light intensity at the surface of culture vessels was 490 µmol/m^2^·s.

The attainment of CP was evaluated in aliquots taken hourly from a synchronous culture aerated at 30°C in the dark. At the end of the cell cycle, the proportions of binuclear daughter cells, 4- and 8-celled daughter coenobia, and undivided mother cells were assessed and plotted against time of darkening. The “commitment curves” illustrated the course of attainment of individual CPs during the cell cycle in synchronous culture [16], [18], [77]. Similarly, percentages of mono-, bi-, tetra- and octonuclear cells were counted in the samples where nuclei were stained by SYBR Green I dye (Molecular Probes) [78]. The proportions of undivided mother cells, mother cells divided into 2, 4 or 8 protoplasts, respectively, and daughter coenobia were determined in samples fixed in 0.25% (w/v) glutaraldehyde and plotted as a function of time.

Cell (coenobium) volume was measured using a Multisizer 3 (Beckman Coulter, www.beckman.com) in samples fixed in glutaraldehyde (0.25% final concentration) by diluting 50 µl of fixed cells into 10 ml of 0.9% (w/v) NaCl; at least 200 cells were gated at the median size.

Caffeine (Fluka, www.sigmaaldrich.com) was added to the specified final concentration from a 100 mM stock solution, zeocin (Duchefa, www.duchefa.com) was added from a 100 mg/ml stock solution, 5-fluorodeoxyuridine was added to the final concentration of 25 mg/l.

### Determination of total DNA and RNA amount

Total nucleic acids were extracted according to Wanka [79], as modified by Decallonne and Weyns [80]. The DNA assay was carried out as described by Decallonne and Weyns [81], with the modifications of Zachleder [82] (see also [83]). Statistical comparison of the data was done by the unpaired Student's t-test between untreated and analyzed samples.

### Kinase assay and western blotting

#### Preparation of protein lysates

Cell pellets containing 2×10^7^ cells were harvested during the cell cycle, washed with SCE buffer (100 mM sodium citrate, 2.7 mM EDTA-Na_2_, pH 7 (citric acid)), snap frozen and stored at −70°C. Protein extracts were prepared as described previously [84].

### Affinity purification of CDKs on CrCKS1 beads, immunoprecipitation of CDKB

To purify CDKs, 20 µl of protein lysate were diluted ten times with RIPA buffer (50 mM HEPES, pH 7.5, 150 mM NaCl, 5 mM EDTA, 5 mM EGTA, 0.1% (w/v) SDS, 1% (v/v) NP-40) containing 1× protease inhibitor cocktail (Sigma P9599, www.sigmaaldrich.com), 1 mM Na_3_VO_4_, 1 mM benzaminidine, 10 mM NaF and incubated for 2 hours at 4°C with 20 µl of 50% w/v CrCKS1 beads slurry. To immunoprecipitate CDKB, 20 µl of the protein lysate were diluted as above and incubated for 1 hour at 4°C with 5 µl of the specific antibody, then 20 µl of 50% w/v slurry of protein A agarose macrobeads (Sigma P1925, www.sigmaaldrich.com) were added and incubated for another 30 minutes at 4°C. Unbound proteins were washed out by 4 consecutive washes with RIPA buffer and 2 washes with kinase buffer (mM HEPES (pH 7.5), 15 mM MgCl_2_, 5 mM EGTA, 1 mM DTT) [85], [86].

Kinase activity was measured according to Bisova et al. [84]. Phosphorylated histone bands were visualized by autoradiography.

### Western blotting

For western blotting, the cleared protein lysates were mixed with 5× SDS-PAGE sample buffer (250 mM Tris-HCl (pH 6.8), 50% (w/v) glycerol, 10% SDS, 100 mM dithiothreitol, 0.5% (w/v) bromophenol blue), incubated 5 min at 65°C and separated by 12% SDS-PAGE [87]. Following the separation, proteins were transferred onto a PVDF membrane (pore size 0.45 µm, Immobilon-P, Millipore, www.millipore.com) [88] at 1 mA/cm^2^ for 1.5 h. The membrane was blocked in 5% (w/v) non-fat dry milk solution in TBS-T buffer (20 mM Tris pH 7.5, 0.5 M NaCl, 0.05% (v/v) Tween 20), overnight at 4°C. The immunodetection was carried out according to standard procedures of the manufacturer. Immunoreactive bands were detected by chemiluminescence (Pierce ECL western blotting substrate, Pierce, www.piercenet.com). The following primary antibodies were used: anti-CDKA1 rabbit antiserum (diluted 1∶1000) raised against a PDFKDTFPKWRPQNC peptide of *C. reinhardtii* CDKA1 protein (Genscript, www.genescript.com), anti-CDKB1 rabbit antiserum (diluted 1∶1000) raised against a QDLHRIFPSLDDSGC peptide of *C. reinhardtii* CDKB1 protein (Genscript, www.genescript.com), and anti-WEE1 rabbit antiserum (diluted 1∶1000) raised against a SQPSQEQYTPDHLTC peptide of *C. reinhardtii* WEE1 protein (Genscript, www.genescript.com). Antibody specificities are described in Results. Secondary antibodies were peroxidase-conjugated goat anti-rabbit IgG (A9169 Sigma, www.sigmaaldrich.com) (diluted 1∶20,000), and peroxidase-conjugated rabbit anti-goat IgG (A5420 Sigma, www.sigmaaldrich.com) (diluted 1∶40,000).

### Microscopy

Observations in transmitted light and fluorescence microscopy were carried out using an Olympus BX51 microscope equipped with a CCD camera (F-ViewII). A U-MWIBA2 filter block (Ex/Em: 460–490/510–550 nm) was used for SYBR Green I fluorescence.

## Supporting Information

Figure S1 Model for Wee1 in the control of the DNA integrity checkpoint in *Arabidopsis thaliana*. DNA stress induced by double-stranded DNA breaks (as induced by γ-irradiation and zeocin) or by blockage of the replication fork (induced by HU and aphidicolin) is sensed mainly by the ATM or ATR signaling cascade, respectively. ATM and ATR simultaneously induce the expression of DNA repair genes and WEE1. WEE1 arrests cells in the G2 phase of the cell cycle, allowing cells to repair DNA before proceeding into mitosis. After de Schutter et al. [14].
(TIF)

Figure S2 Survival of *S. quadricauda* cells on caffeine and zeocin. Plate assay with serially diluted cells spotted on different concentrations of caffeine and zeocin. A 5× serial dilution is presented. The approximate concentrations of cells in each spot are indicated above; the concentrations of the drugs are indicated at the side of each strip.
(TIF)

Figure S3 Protein levels of CDKA (**A**, **B**), CDKB1 (**C**, **D**) and WEE1 (**E**, **F**) kinases in synchronized populations of *S. quadricauda* cells. The cells were grown in the absence or presence of caffeine, zeocin or FdUrd and their combinations from the beginning of the cell cycle as indicated. The upper panels depict protein levels, and bottom panels represent a portion of Ponceau S stained membrane (**A**) or non-specific 95 kDa band detected by anti WEE antibody (**B**) as a loading control. The loading controls are the same for **B** and **C**. An equal volume of protein extract per cell was loaded. A schematic representation of cell cycle progression in the untreated culture is indicated on the top. A representative image of 2 experiments is presented.
(TIF)

Figure S4 Kinase activities of anti CDKA (**A**) and anti CDKB (**B**) immunoprecipitated kinases in synchronized populations of *S. quadricauda* cells. The cells were grown in the absence or presence of caffeine, zeocin or FdUrd and their combinations from the beginning of the cell cycle as indicated. Upper panels depict kinase activities towards histone H1 as a substrate, bottom panels represent Commassie brilliant blue stained histone H1 as a loading control. A schematic representation of cell cycle progression in the untreated culture is indicated on the top. A representative image of 2 experiments is presented.
(TIF)
