# Supplementary figures and images for: DNA Damage during G2 Phase Does Not Affect Cell Cycle Progression of the Green Alga Scenedesmus quadricauda
Source: PLoS One. 2011 May 16;6(5):e19626. doi: 10.1371/journal.pone.0019626 (PMC3095609; doi:10.1371/journal.pone.0019626)

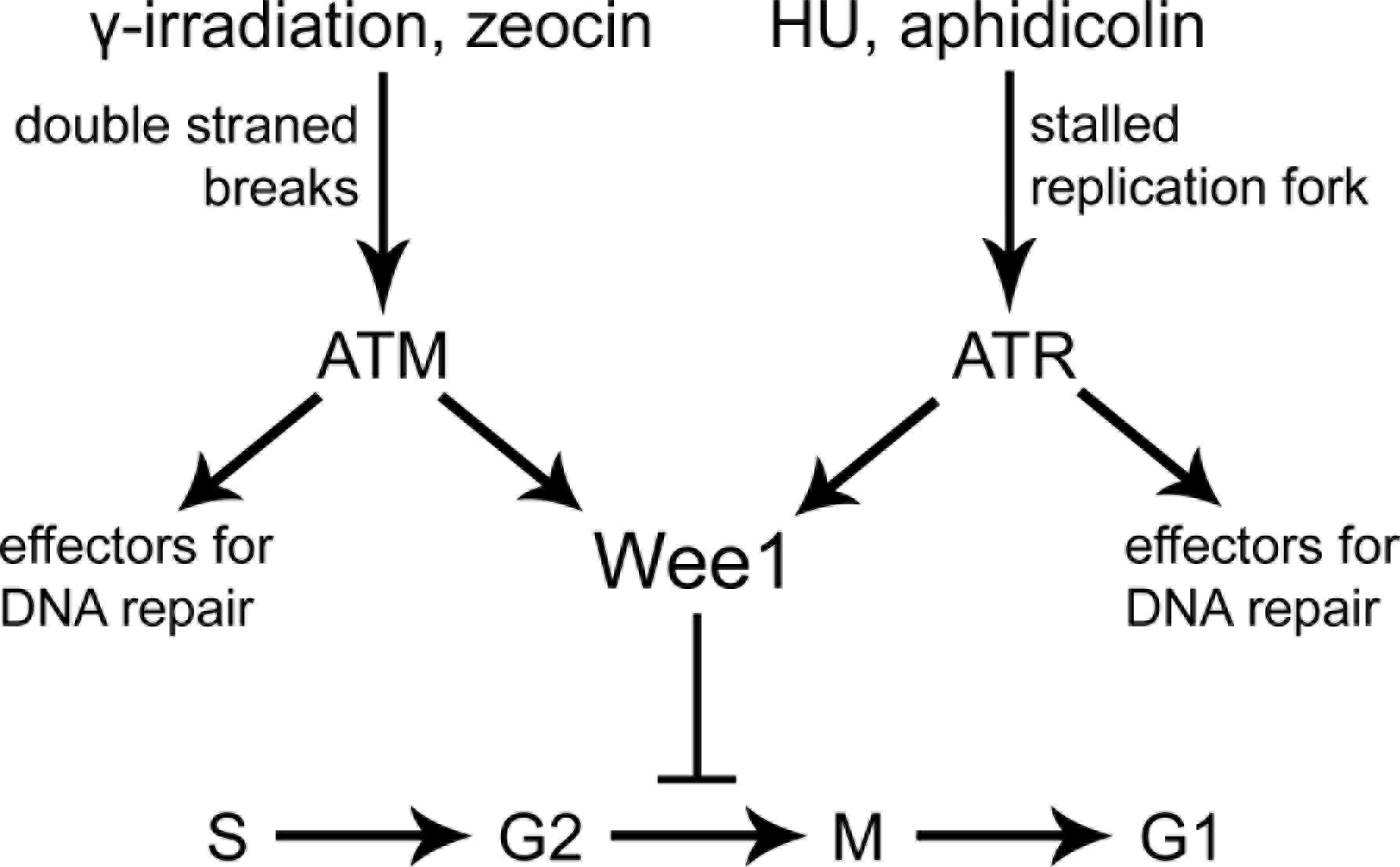

Supplement: Figure S1 — Model for Wee1 in the control of the DNA integrity checkpoint in Arabidopsis thaliana. DNA stress induced by double-stranded DNA breaks (as induced by γ-irradiation and zeocin) or by blockage of the replication fork (induced by HU and aphidicolin) is sensed mainly by the ATM or ATR signaling cascade, respectively. ATM and ATR simultaneously induce the expression of DNA repair genes and WEE1. WEE1 arrests cells in the G2 phase of the cell cycle, allowing cells to repair DNA before proceeding into mitosis. After de Schutter et al. [14]. (TIF) [file pone.0019626.s001.tif]

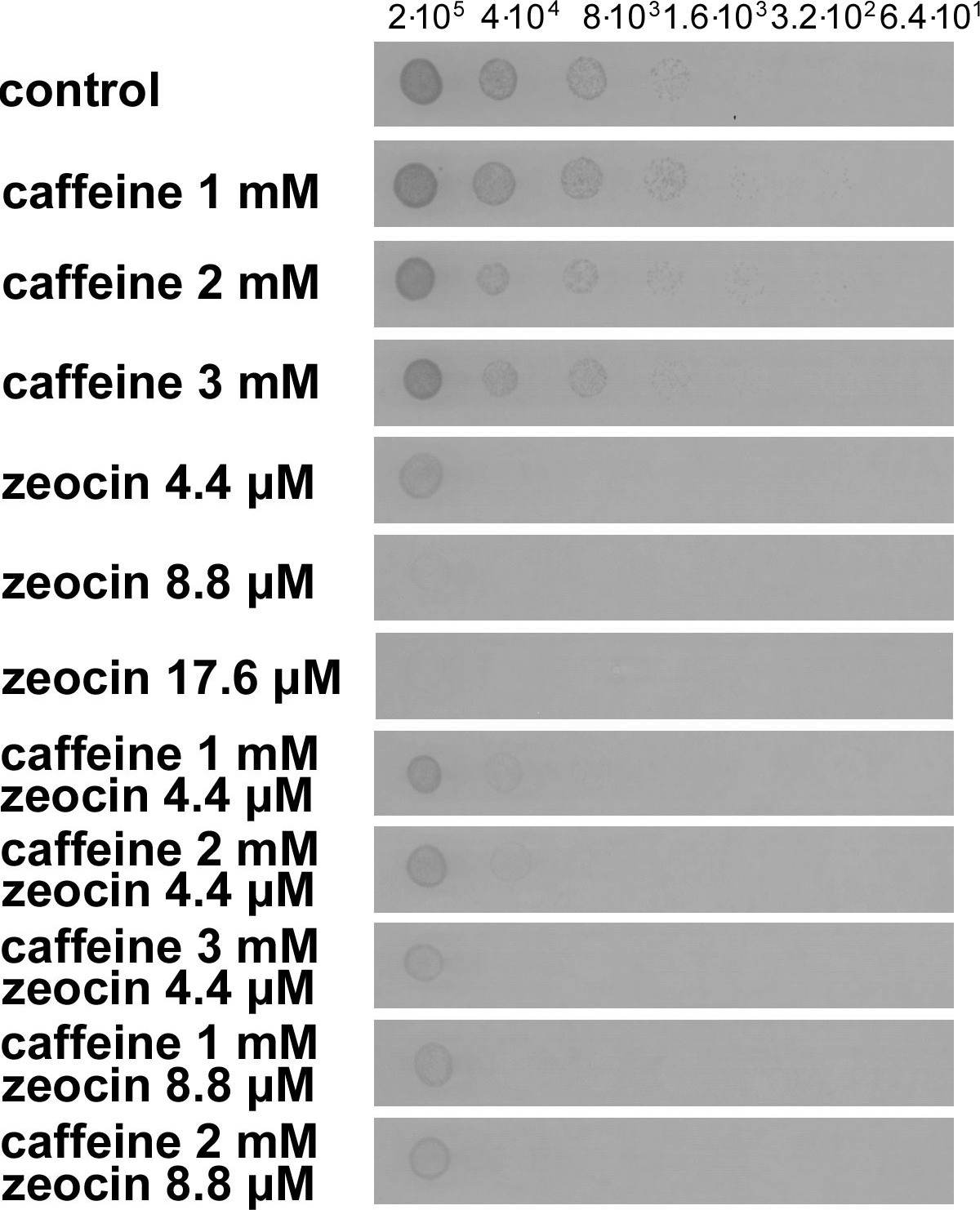

Supplement: Figure S2 — Survival of S. quadricauda cells on caffeine and zeocin. Plate assay with serially diluted cells spotted on different concentrations of caffeine and zeocin. A 5× serial dilution is presented. The approximate concentrations of cells in each spot are indicated above; the concentrations of the drugs are indicated at the side of each strip. (TIF) [file pone.0019626.s002.tif]

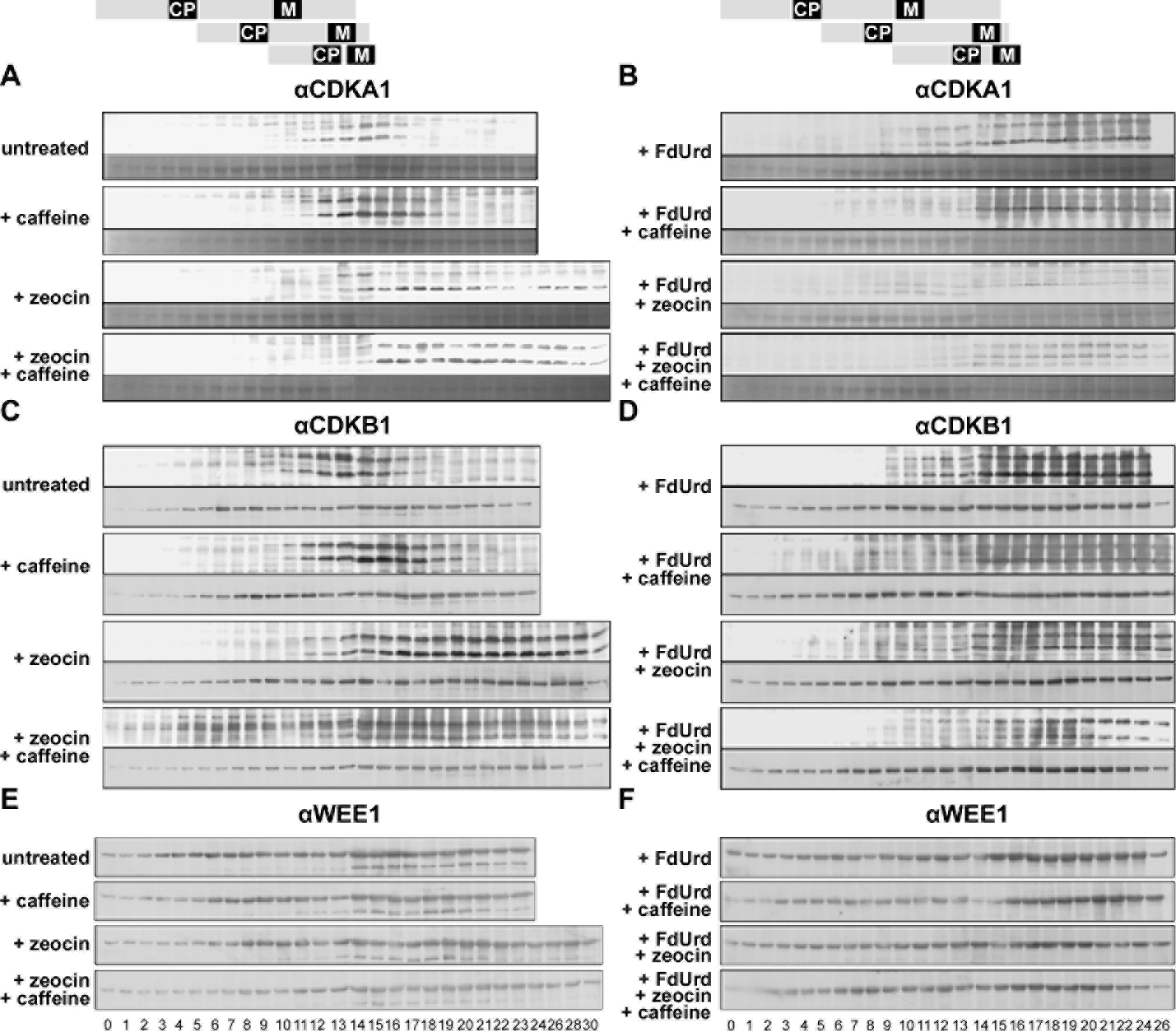

Supplement: Figure S3 — Protein levels of CDKA (A, B), CDKB1 (C, D) and WEE1 (E, F) kinases in synchronized populations of S. quadricauda cells. The cells were grown in the absence or presence of caffeine, zeocin or FdUrd and their combinations from the beginning of the cell cycle as indicated. The upper panels depict protein levels, and bottom panels represent a portion of Ponceau S stained membrane (A) or non-specific 95 kDa band detected by anti WEE antibody (B) as a loading control. The loading controls are the same for B and C. An equal volume of protein extract per cell was loaded. A schematic representation of cell cycle progression in the untreated culture is indicated on the top. A representative image of 2 experiments is presented. (TIF) [file pone.0019626.s003.tif]

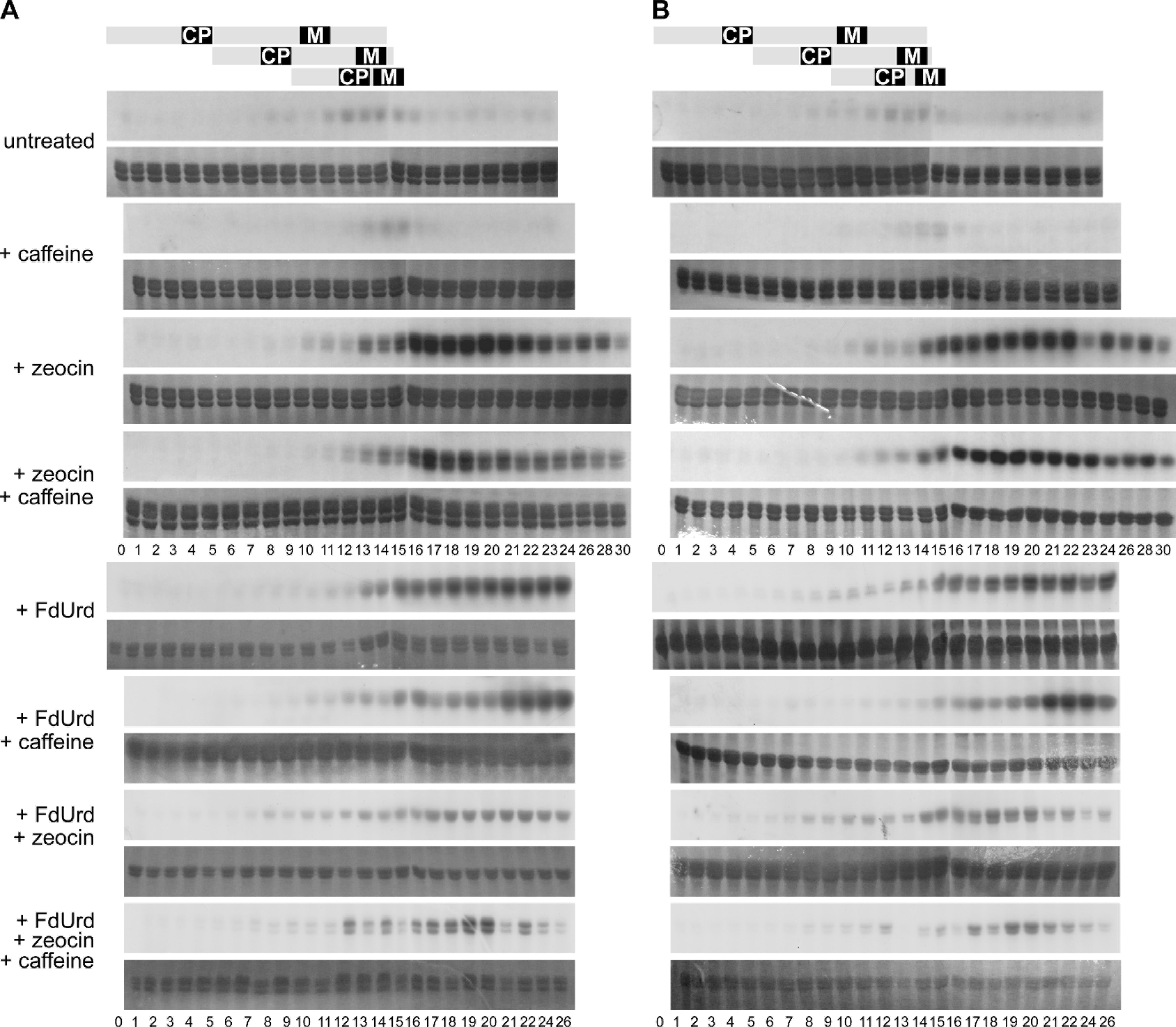

Supplement: Figure S4 — Kinase activities of anti CDKA (A) and anti CDKB (B) immunoprecipitated kinases in synchronized populations of S. quadricauda cells. The cells were grown in the absence or presence of caffeine, zeocin or FdUrd and their combinations from the beginning of the cell cycle as indicated. Upper panels depict kinase activities towards histone H1 as a substrate, bottom panels represent Commassie brilliant blue stained histone H1 as a loading control. A schematic representation of cell cycle progression in the untreated culture is indicated on the top. A representative image of 2 experiments is presented. (TIF) [file pone.0019626.s004.tif]
